# Pericardial Effusion as a Presentation of Lymphangioleiomyomatosis

**DOI:** 10.7759/cureus.73365

**Published:** 2024-11-10

**Authors:** Martinha Vale, Raquel Azevedo, Soraia Araújo, Sofia Esperança, Ana Oliveira

**Affiliations:** 1 Internal Medicine, Unidade Local de Saúde de Braga, Braga, PRT; 2 Infectious Diseases, Unidade Local de Saúde de Braga, Braga, PRT; 3 Critical Care Medicine, Unidade Local de Saúde de Braga, Braga, PRT

**Keywords:** lymphangioleiomyomatosis, mtor inhibitors, pericardial effusion, sirolimus, sporadic lymphangioleiomyomatosis, vascular endothelial growth factor

## Abstract

Lymphangioleiomyomatosis (LAM) is a rare, systemic neoplastic disease that primarily affects women of childbearing age. The disease can arise sporadically or in association with tuberous sclerosis. It is characterized by the proliferation of abnormal smooth muscle-like cells, leading to cystic lung destruction, accumulation of chylous fluid, and development of abdominal tumors. The main clinical presentations are pneumothorax and progressive dyspnea on exertion. In some patients, the onset of symptoms occurs while on estrogen replacement therapy or during pregnancy. Since most patients present with common lung symptoms, such as fatigue and dyspnea, LAM is often the last diagnosis to consider. Worsening symptoms with the menstrual cycle can help raise this suspicion.

This report describes a case of LAM in which the lack of awareness about this rare disease led to a significant diagnostic delay. A 34-year-old Caucasian woman presented with symptoms of dyspnea and fatigue that began during her second pregnancy, which were initially misinterpreted as obstructive lung disease for three years. As the disease evolved, the development of a pericardial effusion, a rare manifestation of the disease, led to the need to perform computed tomography, revealing multiple thin-walled intrapulmonary cysts with diffuse distribution suggestive of LAM, mediastinal lymphadenopathy, and lymphangioma. Serum vascular endothelial growth factor A levels were normal, and spirometry testing revealed severe airflow obstruction. Based on clinical and imaging findings, a diagnosis of LAM was made.

With this article, we intend to raise awareness for the manifestations of this disease and its relation to hormonal changes and review treatment options.

## Introduction

Lymphangioleiomyomatosis (LAM) is a rare, systemic neoplastic disease characterized by the proliferation of abnormal smooth muscle-like cells. This leads to cystic lung destruction, accumulation of chylous fluid, and the development of abdominal tumors such as angiomyolipomas and lymphangioleiomyomas [1,2]. It primarily affects women of childbearing age and can arise sporadically or in association with the tuberous sclerosis complex (TSC), an autosomal dominant syndrome characterized by cerebral calcifications, mental retardation, seizures, and hamartomatous lesions in various organs [3,4]. In recent years, our understanding of the pathophysiology of LAM has advanced, leading to the development of new diagnostic and prognostic biomarkers [2].

In this report, we present a case of sporadic LAM in a 34-year-old woman. We refer to a typical case of LAM in which the nonspecific symptoms and lack of awareness about this disease caused a significant delay in the diagnosis. Eventually, the appearance of pericardial effusion led to the correct diagnosis.

## Case presentation

**Figure 1:**
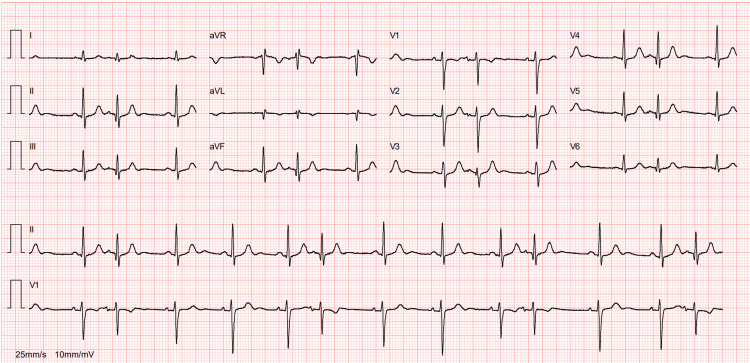
Electrocardiogram revealing sinus rhythm with electrical alternans.

A 34-year-old Caucasian woman with a history of two cesarean sections and breast implants sought medical care due to difficulty breathing during moderate exertion and dry cough, which worsened during menstruation. These symptoms had been progressing for three years, initially appearing during her second pregnancy and significantly worsening over the past two years following a SARS-CoV-2 infection. She has also experienced a 7 kg weight loss in the last 12 months. Initial investigation focused on her respiratory symptoms, as a spirometry test revealed small airway obstructive disease. As a result, she was initially diagnosed with obstructive lung disease and started bronchodilator therapy, but her condition continued to worsen. Functional capacity progressively declined over the next three months, with worsening complaints of dyspnea on minimal exertion, left hemithorax pain causing discomfort in dorsal and left lateral decubitus positions, and paroxysmal nocturnal dyspnea. She then had an electrocardiogram, revealing sinus rhythm with electrical alternans (Figure 1), and a transthoracic echocardiogram that showed a pericardial effusion of less than 20 mm, increased pericardial echogenicity, probable septal bounce, and respiratory variation in mitral flow exceeding 25% and in tricuspid flow exceeding 40%. Given these unexpected findings, she had a cardiology consultation. The echocardiographic study was repeated, confirming a pericardial effusion with a maximum thickness of 20 mm but without criteria for constrictive pericarditis. At this point, she was admitted to the internal medicine department for further investigation.

At physical examination, the patient seemed unimpaired; she presented no signs of respiratory distress, cardiopulmonary auscultation was normal, and she was hemodynamically stable. Blood tests were unremarkable except for a discreet leukocytosis (white blood cell count of 11,800/µL; normal range = 4,000-11,000/µL) with neutrophilia (81%). Arterial blood gas analysis did not reveal hypoxemia.

**Figure 2:**
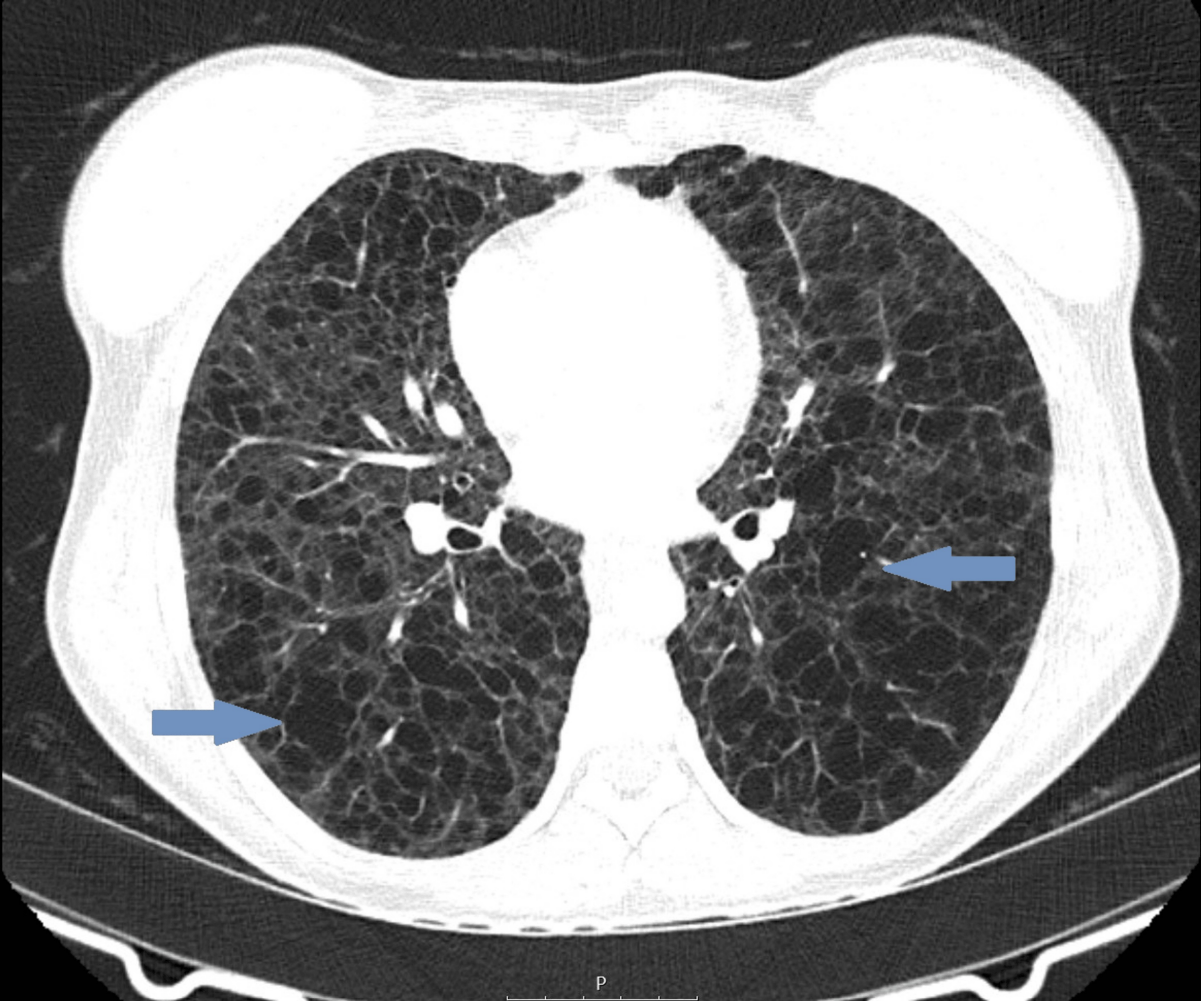
Axial view of thoracic computed tomography scan showing multiple thin-walled intrapulmonary cysts with a diffuse distribution, suggestive of lymphangioleiomyomatosis (arrows).

**Figure 3:**
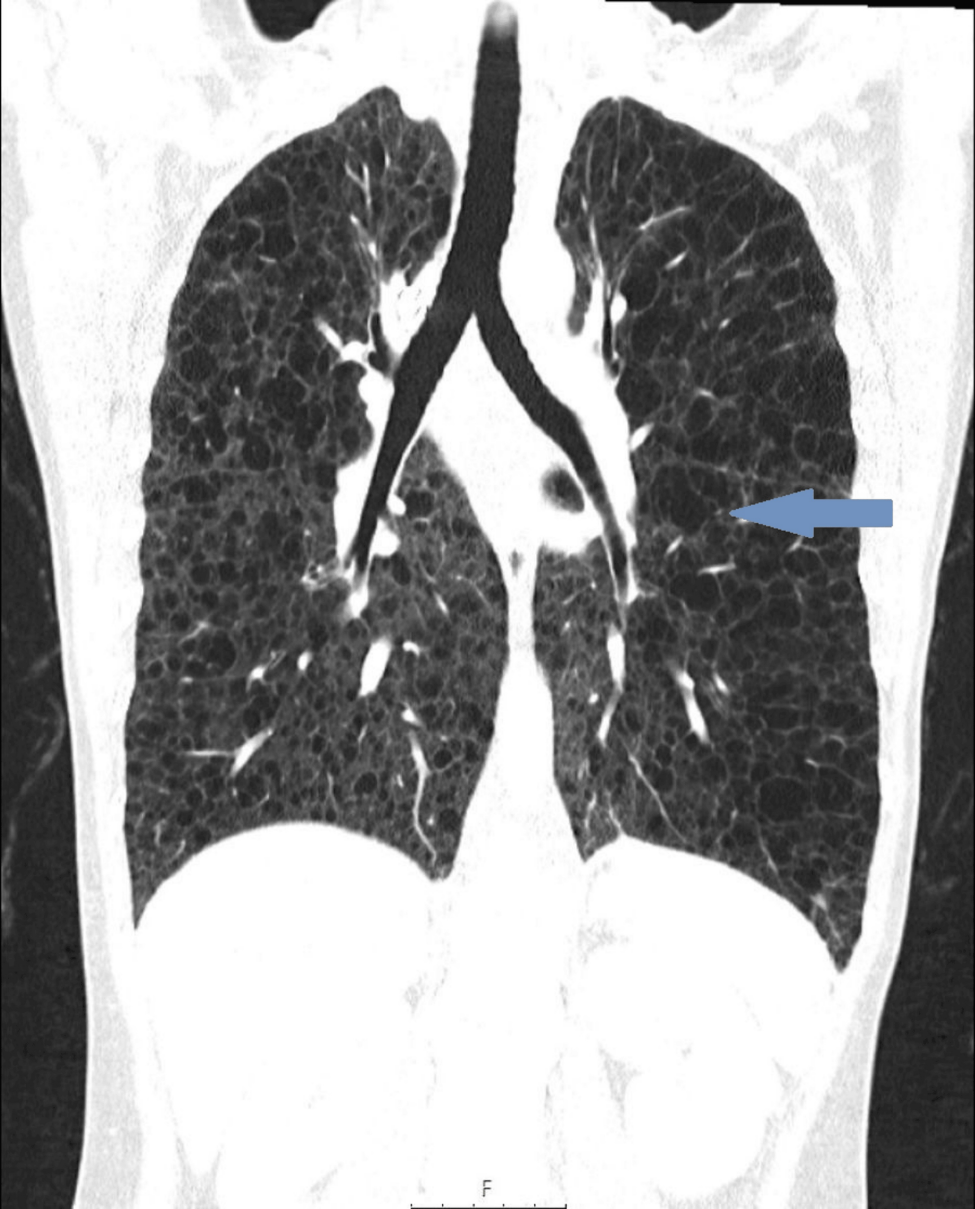
Thoracic computed tomography scan showing diffuse intrapulmonary cysts in coronal view (arrow).

**Figure 4:**
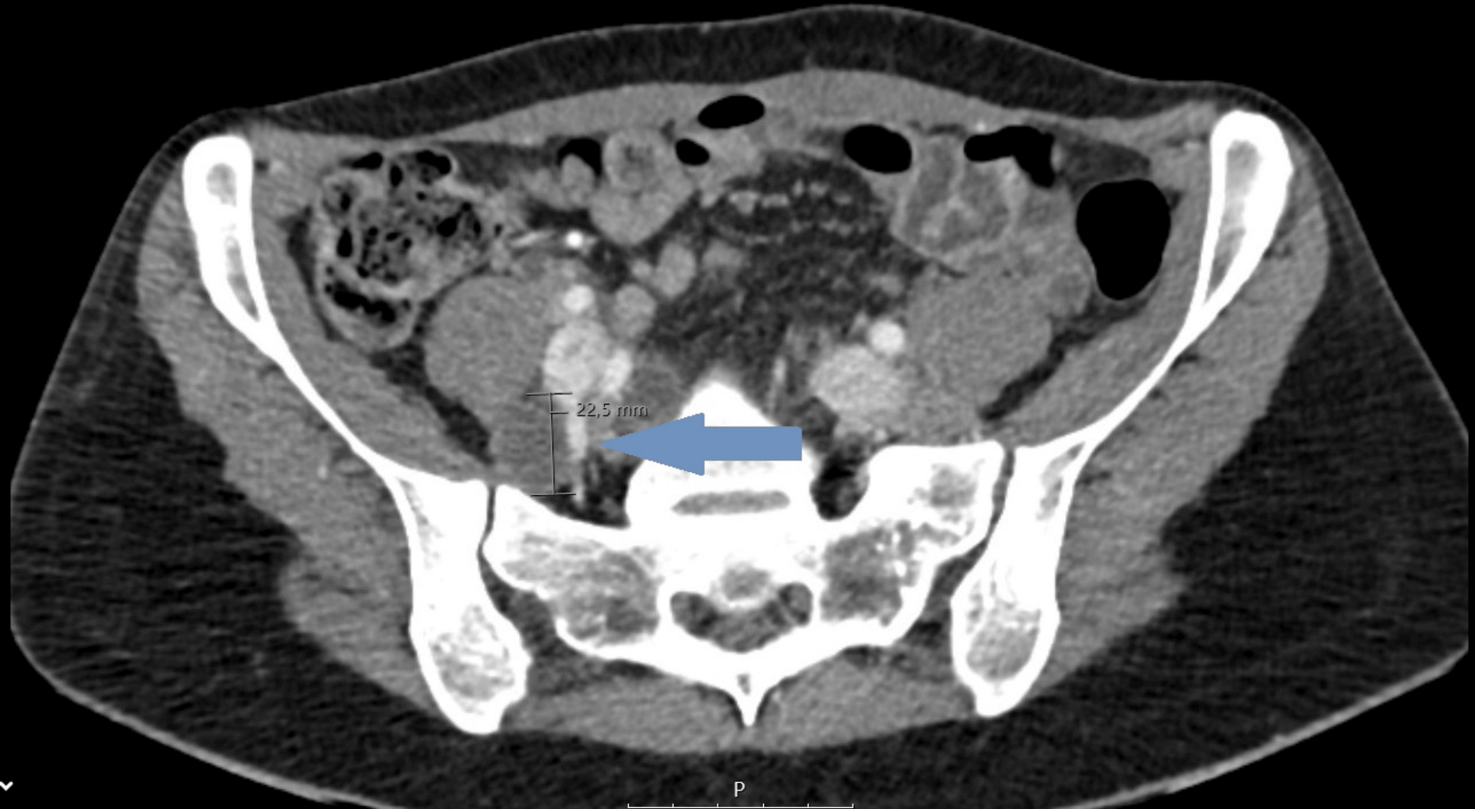
Abdominopelvic computed tomography scan showing a multiloculated cystic formation measuring 78 x 22.5 mm in the upper left retroperitoneum, suggestive of a lymphangioma (arrow).

A computed tomography scan of the chest, abdomen, and pelvis identified multiple thin-walled intrapulmonary cysts with diffuse distribution suggestive of LAM (Figures 2, 3), hypoattenuating mediastinal lymphadenopathies, the largest being intratracheal with 15 mm, and a multiloculated cystic formation measuring 78 x 22.5 mm in the upper left retroperitoneum, suggestive of a lymphangioma, was also present (Figure 4). Serum vascular endothelial growth factor (VEGF)-D level was normal (26 pg/ml; normal range < 115 pg/ml). Spirometry testing revealed severe airflow obstruction, with a forced expiratory volume in one second (FEV1) of 40% and a negative bronchodilator response.

Based on clinical and imagiological findings, the diagnosis of LAM was made, and immunosuppressive therapy with sirolimus (2 mg once daily) was instituted. However, three months after treatment, she maintained exertional dyspnea, and severe airflow obstruction persisted. Diffusing capacity for carbon monoxide (DLCO) testing elicited deficient diffusing capacity of the lungs; the six-minute walk test showed significant oxygen desaturation, with a 22% drop and a distance of 574 meters covered. Due to the lack of clinical improvement despite medical treatment, she will soon be considered for lung transplantation.

## Discussion

LAM occurs almost exclusively in women of reproductive age, with an incidence of about five cases per million [1,5,6]. Sporadic LAM affects one in 400,000 adult females; in TSC, LAM occurs in 30-40% of adult females but has also been reported in males and children [3]. There is no ethnic or geographic variability in the incidence of LAM [7]. Both forms are caused by mutations in the tuberous sclerosis genes TSC1 and TSC2, resulting in the loss of function of their protein products hamartin and tuberin, respectively; this leads to inappropriate signaling through the mammalian target of rapamycin (mTOR) pathway, with its constitutive activation, promoting cell growth and proliferation [2,4]. LAM cells increase the expression of angiogenic VEGF-A and lymphangiogenic growth factors VEGF-C and VEGF-D, facilitating their spread to the circulation and the degradation of extracellular matrix, which leads to cyst formation [2,7].

Female hormones play an important role in the development of the disease, as lung function is known to worsen during periods of high levels of circulating estrogen, such as pregnancy, menstruation, and under hormonal contraception [2,5,7]. This is in line with our case in which the patient started complaining of dyspnea during pregnancy and noticed worse respiratory symptoms through menstruation. In the post-menopausal period, patients with LAM often experience disease stabilization [2,5,7].

The main clinical presentations of LAM are pneumothorax and progressive dyspnea on exertion. However, those are often misinterpreted as asthma or chronic obstructive pulmonary disease, leading to a delay in diagnosis of three to five years [7]. The most common functional abnormalities are airflow obstruction, which is present in 25-66% of cases, and decreased DLCO in 90% of cases [2,4,7]. Our case highlights the diagnostic challenge, as the patient was initially diagnosed with obstructive lung disease, leading to a significant delay in the correct diagnosis. A spontaneous pneumothorax in a premenopausal woman should raise suspicion of LAM, as one-third of patients will present with a pneumothorax, and 50-80% of patients will have a pneumothorax in the course of the disease [2,7]. Pericardial effusion as a result of LAM is uncommon, but it ultimately led to the diagnosis in our patient. Other symptoms described in the literature are hemoptysis, cough, chest pain, and chylothorax [2,7].

LAM is also characterized by other extrapulmonary manifestations, namely, renal angiomyolipomas and lymphatic involvement [2]. In our case, lymphangioma was identified as part of the patient's extrapulmonary manifestations, reinforcing the multisystemic nature of the disease. Angiomyolipomas are benign tumors of smooth muscle, blood vessels, and fat that are present in more than 80% of women with TSC-LAM and around 30-40% of sporadic cases [2]. Most of them are found in the kidneys and are small and asymptomatic but can complicate with bleeding and abdominal pain [2]. Lymphatic manifestations are much more common in sporadic LAM [2]. Lymphangioleiomyomas are lymphatic cystic masses observed in 16-38% of LAM patients and can be asymptomatic or result in abdominal pain, bloating, edema of lower extremities, or urinary symptoms [2]. Lymphadenopathy is present in 25-77% of patients with LAM, often in the pelvis or retroperitoneum [2]. Chylothorax is the most common pleural effusion in LAM, reported in 10-30% of cases; it is caused by disruption or blockage of the thoracic duct or one of its branches by neoplastic LAM cells [2].

A thoracic high-resolution CT (HRCT) scan showing diffuse and thin-walled pulmonary cysts is the hallmark of the disease [2,5,7]. In our patient, thoracic CT confirmed the presence of diffuse intrapulmonary cysts as well as lymphadenopathies. The cysts are formed due to smooth muscle cell proliferation; they are characteristically round, well-defined, devoid of internal structure, relatively uniform, measuring 2-30 mm, widely distributed throughout the lungs, and without lobar predominance [2,5,7]. Nevertheless, a formal diagnosis requires at least one other accompanying feature: the diagnosis of TSC, renal angiomyolipomas, elevated VEGF-D (≥800 pg/mL), a chylous effusion, or a lymphangioleiomyoma [7,8]. VEGF-D is elevated in 70% of cases of LAM and has diagnostic and prognostic value [7]. The identification of lymphangioleiomyomas by imagiological characteristics is attainable due to the presence of fat in the tumors. Other abnormalities that can be found in thoracic HRCT include chylothorax, pneumothorax, pulmonary hemorrhage, ground-glass opacities, septal thickening, thoracic duct dilatation, and mediastinal lymph node enlargement [2,7,8].

Lung biopsy should be reserved for cases with parenchymal cysts on HRCT that are characteristic of LAM in the absence of additional clinical, radiologic, or serologic features [8]. Histologically, the main features of pulmonary LAM are lung cysts and LAM cells. These cells have smooth muscle features, which are identified by anti-actin and anti-desmin staining [4,7,9]. They also express melanoma-associated antigens, which are detected by HMB-45 staining [4,7,9]. The estrogen and progesterone receptors may likewise help the diagnosis [4,7,9].

Pulmonary function tests are unspecific in LAM but are useful for disease stratification and assessing response to treatment [2,4,7]. The worsening of airflow obstruction and the decline of DLCO both correlate with disease severity [2,4,7].

The primary treatment for parenchymal lung disease due to sporadic LAM is sirolimus, an inhibitor of the mTOR signaling pathway, which has been shown to reduce loss of lung function, chylous accumulation, and angiomyolipoma growth [7,10]. According to the American Thoracic Society and Japanese Respiratory Society guidelines, sirolimus is indicated for patients with abnormal lung function, defined as FEV1 less than 70% of predicted, patients with rapidly progressive disease, or with symptomatic chylous fluid effusions [1,7]. Inhaled bronchodilators should be considered for symptomatic relief, especially in patients with obstructive airway disease [7]. Although the association between LAM and estrogen is established, studies with antihormonal therapies have not shown efficacy and, therefore, are not recommended [7]. Ongoing trials with interferon-gamma, statins, hydroxychloroquine, anti-estrogen-tamoxifen, prostaglandin inhibitors, and tyrosine kinase inhibitors are in progress [7,11-13]. Lung transplantation is an option for patients with advanced LAM or for those with no improvement with mTOR inhibitor treatment, entailing a 52-55% chance of survival after 10 years [7,14]. Despite sirolimus therapy, the patient did not show significant improvement, a common finding as treatment response may be partial, especially in cases of advanced or progressive disease, as discussed in the literature [7,10]. The lack of response to sirolimus treatment was an important consideration in evaluating the patient for lung transplantation. Supportive care measures include smoking cessation, influenza, pneumococcal and COVID-19 vaccination, pulmonary rehabilitation, and supplemental oxygen when indicated. It is also recommended to refer the patient to a specialized center.

## Conclusions

The prevalence of LAM is estimated to be much higher than reported, as many patients remain asymptomatic or misdiagnosed. This case highlights the importance of being aware of this diagnosis, especially when faced with a woman of reproductive age with unexplained dyspnea, spontaneous pneumothorax, hemoptysis, or abnormal chest radiograph. The significant variability of how LAM can manifest is well demonstrated in our article, but symptoms in relation to hormonal changes should always raise suspicion of LAM.

Sirolimus is the mainstay of treatment, as studies suggest that maintaining therapy hampers the progression of the disease. Future advances in understanding the physiopathological mechanisms of LAM will be critical to the development of new therapies and prognostic markers that can change the natural history of the condition.

## References

[1] McCormack FX, Gupta N, Finlay GR (2016). Official American Thoracic Society/Japanese Respiratory Society clinical practice guidelines: lymphangioleiomyomatosis diagnosis and management. Am J Respir Crit Care Med.

[2] O'Mahony AM, Lynn E, Murphy DJ, Fabre A, McCarthy C (2020). Lymphangioleiomyomatosis: a clinical review. Breathe (Sheff).

[3] Johnson SR, Cordier JF, Lazor R (2010). European Respiratory Society guidelines for the diagnosis and management of lymphangioleiomyomatosis. Eur Respir J.

[4] Torre O, Elia D, Caminati A, Harari S (2017). New insights in lymphangioleiomyomatosis and pulmonary Langerhans cell histiocytosis. Eur Respir Rev.

[5] Cong CV, Anh TT, Ly TT, Duc NM (2022). Pulmonary lymphangioleiomyomatosis (LAM): a literature overview and case report. Radiol Case Rep.

[6] Shah JM, Patel JT, Shah H, Dadigiri H, Alla A, Cheriyath P (2023). The epidemiology and clinical features of lymphangioleiomyomatosis (LAM): a descriptive study of 33 case reports. Cureus.

[7] McCarthy C, Gupta N, Johnson SR, Yu JJ, McCormack FX (2021). Lymphangioleiomyomatosis: pathogenesis, clinical features, diagnosis, and management. Lancet Respir Med.

[8] Gupta N, Finlay GA, Kotloff RM (2017). Lymphangioleiomyomatosis diagnosis and management: high-resolution chest computed tomography, transbronchial lung biopsy, and pleural disease management. An official American Thoracic Society/Japanese Respiratory Society clinical practice guideline. Am J Respir Crit Care Med.

[9] Klarquist J, Barfuss A, Kandala S (2009). Melanoma-associated antigen expression in lymphangioleiomyomatosis renders tumor cells susceptible to cytotoxic T cells. Am J Pathol.

[10] McCormack FX, Inoue Y, Moss J (2011). Efficacy and safety of sirolimus in lymphangioleiomyomatosis. N Engl J Med.

[11] El-Chemaly S, Taveira-DaSilva A, Bagwe S (2020). Celecoxib in lymphangioleiomyomatosis: results of a phase I clinical trial. Eur Respir J.

[12] Krymskaya VP, Courtwright AM, Fleck V, Dorgan D, Kotloff R, McCormack FX, Kreider M (2020). A phase II clinical trial of the safety of simvastatin (SOS) in patients with pulmonary lymphangioleiomyomatosis and with tuberous sclerosis complex. Respir Med.

[13] El-Chemaly S, Taveira-Dasilva A, Goldberg HJ (2017). Sirolimus and autophagy inhibition in lymphangioleiomyomatosis: results of a phase I clinical trial. Chest.

[14] Khawar MU, Yazdani D, Zhu Z, Jandarov R, Dilling DF, Gupta N (2019). Clinical outcomes and survival following lung transplantation in patients with lymphangioleiomyomatosis. J Heart Lung Transplant.

